# Two Enhancers Control Transcription of *Drosophila muscleblind* in the Embryonic Somatic Musculature and in the Central Nervous System

**DOI:** 10.1371/journal.pone.0093125

**Published:** 2014-03-25

**Authors:** Ariadna Bargiela, Beatriz Llamusi, Estefanía Cerro-Herreros, Ruben Artero

**Affiliations:** 1 Translational Genomics Group, Department of Genetics, University of Valencia, Valencia, Spain; 2 INCLIVA Health Research Institute, Valencia, Spain; Alexander Fleming Biomedical Sciences Research Center, Greece

## Abstract

The phylogenetically conserved family of Muscleblind proteins are RNA-binding factors involved in a variety of gene expression processes including alternative splicing regulation, RNA stability and subcellular localization, and miRNA biogenesis, which typically contribute to cell-type specific differentiation. In humans, sequestration of Muscleblind-like proteins MBNL1 and MBNL2 has been implicated in degenerative disorders, particularly expansion diseases such as myotonic dystrophy type 1 and 2. *Drosophila muscleblind* was previously shown to be expressed in embryonic somatic and visceral muscle subtypes, and in the central nervous system, and to depend on Mef2 for transcriptional activation. Genomic approaches have pointed out candidate gene promoters and tissue-specific enhancers, but experimental confirmation of their regulatory roles was lacking. In our study, luciferase reporter assays in S2 cells confirmed that regions P1 (515 bp) and P2 (573 bp), involving the beginning of exon 1 and exon 2, respectively, were able to initiate RNA transcription. Similarly, transgenic *Drosophila* embryos carrying enhancer reporter constructs supported the existence of two regulatory regions which control embryonic expression of *muscleblind* in the central nerve cord (NE, neural enhancer; 830 bp) and somatic (skeletal) musculature (ME, muscle enhancer; 3.3 kb). Both NE and ME were able to boost expression from the Hsp70 heterologous promoter. In S2 cell assays most of the ME enhancer activation could be further narrowed down to a 1200 bp subregion (ME.3), which contains predicted binding sites for the Mef2 transcription factor. The present study constitutes the first characterization of *muscleblind* enhancers and will contribute to a deeper understanding of the transcriptional regulation of the gene.

## Introduction

Muscleblind proteins were initially identified in *Drosophila* and associated to the development of the embryonic peripheral nervous system [1], the muscles [2] and the adult photoreceptors [3]. They were later found to regulate alternative splicing of defined pre-mRNAs by binding to specific consensus sequences and to hairpins containing pyrimidine mismatches through conserved zinc finger motifs of the CCCH type ([4], [5] and reviewed in [6]). Muscleblind target transcripts encode cell adhesion and cytoskeleton components, proteins involved in muscle excitation and contraction, structural proteins in muscle sarcomere and signalling molecules, among others [5], [7], [8], [9], [10], [11]. Through alternative splicing *musclebIind* transcripts themselves generate at least fourteen transcript isoforms. Most of them share common 5′ sequences but differ at the 3′-ends, encoding proteins of different lengths and carboxyl termini. The *muscleblind* transcriptional unit is large and has a complex organization with ten exons distributed over about thirty times more than the average gene length in *Drosophila* [5], [12].

In contrast to *Drosophila*, which has a single gene, three Muscleblind-like homologs (*MBNL1*, *MBNL2*, and *MBNL3*) exist in humans and mice [13], [14]. Although recent results have highlighted MBNL proteins as regulators of messenger RNA (mRNA) stability [15], [16], [17], localization [10], [18] or miRNA biogenesis [19] in the cytoplasm, these proteins are particularly well-known for their nuclear function as alternative splicing regulators. MBNL1 plays a primary role in alternative splicing allowing the fetal-to-adult splicing transitions needed for development of skeletal and cardiac muscle whereas MBNL2 seems to perform a similar function in the central nervous system [20], [21]. Similarly, MBNL1 and MBNL2 are direct negative regulators of a large program of cassette exon alternative splicing events that are differentially regulated between embryonic stem cells and other cell types [22]. In contrast, MBNL3 has been reported as a member of the family with unusual functions. MBNL3 antagonizes muscle differentiation by promoting exclusion of the alternatively spliced β-exon of *Myocyte enhancer factor 2D* (*Mef2D*) [23] and also by the inhibition of myogenesis by maintaining myoblasts in a proliferative state [24], [25]. As a result of this regulation a negative correlation exists between MBNL1 and MBNL3 expression levels in muscle during development when MBNL3 is mainly detected during embryonic development, but also transiently during injury-induced adult skeletal muscle regeneration [13], [26]. MBNL1 and MBNL2 have a similar expression pattern in skeletal and heart muscle, kidney, liver, lung, intestine, brain and placenta. However, MBNL1 expression in skeletal muscle is higher than MBNL2 [13], [25].

*Drosophila* Muscleblind shows tissue-specific expression during development. In eye-antennal imaginal discs Muscleblind is required for the formation of photoreceptor rhabdomeres, identifying *muscleblind* as a general factor required for terminal differentiation of adult ommatidia [3]. Its expression was also reported in the embryonic central nervous system and in the somatic muscles, where disruption of *muscleblind* caused defects on muscle attachments to the epidermis and disrupted Z-band formation in muscle sarcomeres [2]. Recent studies have revealed a role for *muscleblind* in the myoblast fusion process through a splice-independent regulation of *muscle protein 20* (*Mp20*), a gene that promotes myoblast fusion [11]. Consistent with its function during terminal muscle differentiation, *Drosophila* Myocyte enhancer factor 2 (Mef2) activates *muscleblind* in embryos, placing this gene downstream of *Mef2* function in the myogenic differentiation program in flies [2]. The *muscleblind chaste* mutation has revealed that the gene is not only required during embryo development but also in adult brain, where it is necessary for the normal development of neural circuitry that regulate female sexual receptivity [27].

Muscleblind-like proteins are critically involved in many pathogenesis pathways, but most notably in myotonic dystrophies type 1 and type 2 (DM1 and DM2; reviewed in [6], [28]). DM1 is caused by the expansion of the unstable CTG triplet in the 3′untranslated region of the *Dystrophia Myotonica Protein Kinase* (*DMPK)* gene [29]. DM2 patients carry an unstable CCTG repeat expansion in intron 1 of *CCCH-type zinc finger nucleic acid binding protein* (*CNBP*) [30]. In both cases, transcribed repeat expansions form ribonuclear foci that have the ability to sequester, among others, MBNL proteins, which are therefore depleted from their normal functions [14], [31], [32]. DM1 and DM2 are typically regarded as muscular diseases but many other organs are also affected resulting in eye cataracts, cognitive dysfunction and cardiac conduction defects.

Despite biomedical and developmental relevance, the knowledge on the transcriptional regulation of *muscleblind* genes, particularly in *Drosophila* and in humans, is extremely limited. With the aim to fill this gap, in this study we have performed *in silico* and *in vivo* analyses to define gene promoters and tissue-specific *cis*-regulatory regions that control *Drosophila muscleblind* expression. Using a candidate approach, we have identified two putative gene promoters, located in exon 1 and exon 2, and have confirmed two intronic regions with the ability to drive expression to embryonic somatic muscle and the nerve cord. This constitutes the first description of tissue-specific enhancers and provides new insights into the *muscleblind* gene.

## Results

### 1. Mapping of the Promoter Regions of *Drosophila muscleblind*

**Figure 1:**
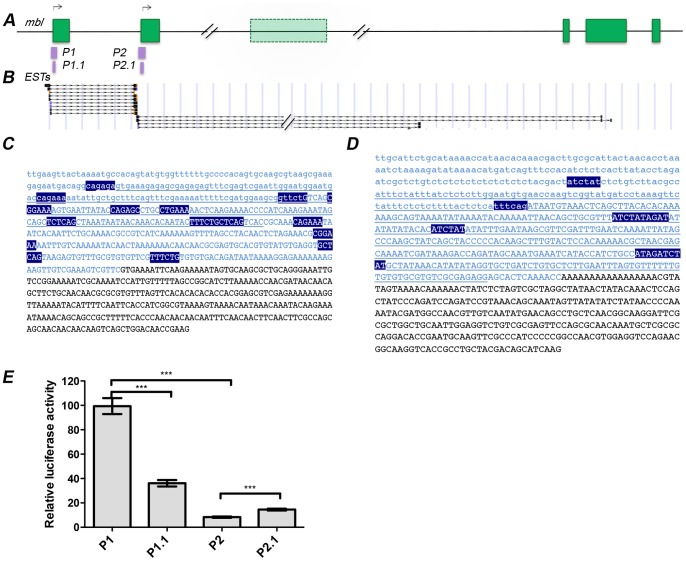
Organization of the *muscleblind* genomic region. (A) Representation of 90 kb of the *Drosophila melanogaster muscleblind* gene. Green boxes represent exons and black lines introns. Representation according to [5]. Candidate promoter regions P1 and P2, and their shorter versions P1.1 and P2.1, are indicated. Black arrows denote putative transcription start sites located in exons 1 and 2. (B) 5′-Ends of ESTs mapping to the *muscleblind* gene, according to the UCSC Genome Browser, suggest that most transcripts start in exon 1 and 2. Genomic context around exon 1 (C) and exon 2 (D). Exonic sequence is in capital letters, P1 and P2 are highlighted in blue (GenBank accession numbers KJ398152 and KJ398154, respectively), and P1.1 and P2.1 are underlined (GenBank accession numbers KJ398151 and KJ398153, respectively). Blue boxes denote promoter consensus sequences with Sig value greater than 7 according to Scope (significant by default). (E) Relative luciferase activity from transiently transfected *Drosophila* S2 cells. Luciferase activity was stronger from P1 (or P1.1) than from P2 (or P2.1) promoters. Luciferase activity was measured 48 h after transfection. *Renilla* expression levels were used to normalize cell number, transfection efficiency, and general effects on gene transcription. All data were also normalized to luciferase levels of the empty vector pGL3 Basic. ***P<0.001. Bar graph shows means+s.e.m. from three independent experiments with three technical replicates each.

The analysis of available cDNA sequences and expressed sequence tags (EST) involving the *muscleblind* locus showed that 5′-end sequences clustered to two locations in the gene, to the beginning of exon 1 and to the beginning of exon 2 (Fig. 1B), which suggests that *muscleblind* might have two transcription start sites (TSS). To test this hypothesis we defined two regions as potential promoters of *muscleblind*. P1 ranged from −180 to +335 (515 bp long) while P2 spanned from −243 to +343 (586 bp long) (Fig. 1*A*), defining as +1 the first bp in exon 1 and exon 2, respectively. Although core promoter regions are typically defined as +50 to −50 of transcription start site [33], a longer region was used to include not only the core promoter but also proximal promoter sequences with potential activator binding sites.

A SCOPE database analysis of *Drosophila* promoter elements [34] confirmed the accumulation of known consensus sequences in P1 and P2, thus supporting their potential as promoter regions (Fig. 1 *C,D*). Furthermore, these motifs were phylogenetically conserved among *Drosophila* species in the Multiz Alignments & phastCons Scores provided by the UCSC, supporting the relevance of these non-coding regions (data not shown). To test the functional relevance of the putative promoters we generated reporter constructs in which P1 and P2 drove expression of Firefly luciferase. In addition, we also tested the activity of shorter versions, contained in the longer ones, of 220 bp long (−104 to +116; P1.1) and 397 bp long (−81 to +316; P2.1), respectively, in an attempt to define minimal promoters. Dual luciferase reporter assays in *Drosophila* S2 cells transfected with the resulting constructs revealed that P1 was able to boost luciferase readings more than 100 fold higher relative to the promoter-less control. This was 2.5 fold the transcription measured for P1.1, 12 fold higher than the luciferase activity driven by P2 and 7 times the activity measured for P2.1 (Fig. 1*E*). Thus, robust expression of luciferase was observed in P1 constructs in comparison to reporter expression driven by P2, and the higher activity obtained with P1 in comparison to P1.1 suggests that P1 contains proximal promoter elements that are not included in P1.1.

### 2. Identification of Putative *cis*-regulatory Modules

**Figure 2:**
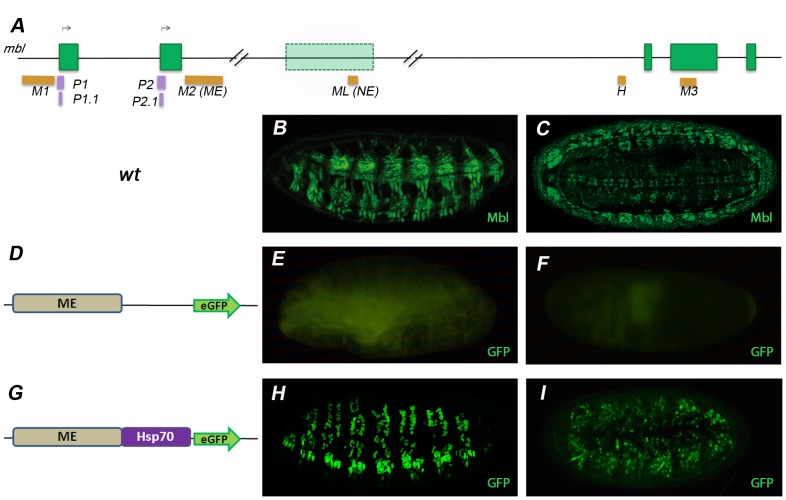
ME reproduces *muscleblind* expression in the embryonic somatic musculature. (A) Localization of the putative *cis*-regulatory modules M1, M2, ML, H and M3, indicated as orange boxes, in the context of the *muscleblind* genomic locus. Fluorescence confocal images of lateral (B,E,H,) and ventral (C,F,I,) views of late *Drosophila* embryos. (D,G) Schematic representation of the reporter constructs used to transform the germline of *Drosophila*. In control *yw* flies (B,C) an anti-Mbl antibody detects robust expression in the somatic musculature and in the CNS (green). Direct visualization of the GFP reporter under the control of the ME enhancer in the pH-Stinger vector (E,F,H,I). Promoter-less ME constructs (D–F) do not activate GFP expression and serve as negative controls. Flies carrying the ME enhancer upstream of *Hsp70* (G–I) reproduce Muscleblind expression in the somatic musculature but not in the CNS. All micrographs were taken at 200× magnification. Anterior is to the left and dorsal up unless otherwise stated.

Potential *cis*-regulatory elements of transcription can be identified as highly conserved non-coding regions in phylogenetic footprinting analyses (as an example see [35]). A fragment of 120 kb harbouring most of the *muscleblind* gene, plus 20 kb upstream of the gene (complete sequence analyzed chr2R: 13133058–13252891), was used as the reference DNA to align orthologous sequences from 12 *Drosophilids*. Using the bioinformatics tool rVista, an intronic sequence showing above 90% identity in a 100 bp window between *Drosophila melanogaster* and *D.mojavensis* was selected. This sequence, refered to as H region, was 872 bp long and was located in intron 2 (Fig. 2*A* and Table 1). Moreover, chromatin immunoprecipitation followed by microarray analysis (ChIP-on-chip) data revealed putative *cis*-regulatory modules (CRMs) M1, M2, M3 and ML (Fig. 2*A* and Table 1) in the *muscleblind* locus that bound *Drosophila* Mef2 [36], [37], [38]. We found these results particularly relevant because Mef2 is a known activator of *muscleblind* expression in the *Drosophila* embryo [2]. Interestingly, these candidate CRMs not only bind Mef2 in ChIP-on-chip experiments but also other muscle organizing factors such as Biniou (Bin), Tinman (Tin) or Twist (Table 1). The ML region only bound Mef2 in late embryos according to [37].

**Table 1:** Putative *cis*-regulatory modules (CRMs) chosen for *in vivo* testing of reporter expression.

CRM	Location	Size (bp)	TF binding sites
**M1**	Upstream	2278	1 Twi
			6 Mef2
			1 Bin
**M2**	Intron 2	3340	2 Mef2
			3 Bin
**M3**	Exon 4	1240	3 Mef2
			1 Bin
**ML**	Intron 2	830	3 Mef2
			1 Bin
**H**	Intron 2	872	0

All except region H according to [37].

### 3. *In vivo* Testing of Reporter Expression Reveals Tissue-specific Enhancers

**Figure 3:**
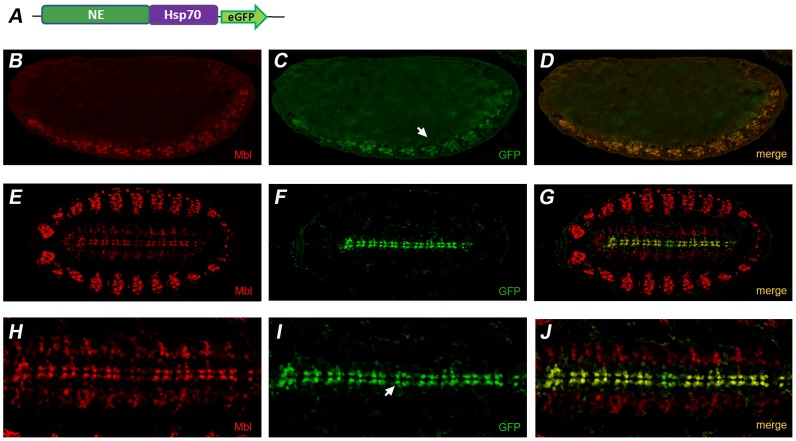
NE reproduces *muscleblind* expression in the central nervous system. (A) Schematic representation of the reporter construct used to transform the *Drosophila* germline. Fluorescence confocal images of lateral (B–D) and ventral (E–J) views of late *Drosophila* embryos expressing construct (A) co-stained with anti-GFP (green) and anti-Mbl antibodies (red). NE drives expression in the CNS (arrows; C,I) overlapping Muscleblind expression (B,E,H; D,G,J shows the merge in yellow). No signal of the reporter was observed in tissues other than CNS. Endogenous Muscleblind expression in the muscles is in focus in (E,G,H,J). Micrographs were taken at 200× (B–G) and 400× magnification (H–J). Anterior is to the left and dorsal up unless otherwise stated.

To test the regulatory potential of the highly conserved (H) and the Mef2-bound (M1, M2, M3 and ML) genomic regions involving the *muscleblind* locus, we generated fusion constructs in the *Drosophila* transformation vector pH-Stinger. This vector contains the *heat shock protein 70* (*Hsp70*) promoter and is specifically designed to avoid chromatin configuration effects (“position effects”) by flanking the eGFP reporter cDNA with two copies of insulator sequences from the *gypsy* transposon [39]. Embryonic expression of the eGFP was assessed either directly (green fluorescence) (Fig. 2) or immunodetecting the reporter protein with a polyclonal anti-GFP antibody (Fig. 3). Only embryos carrying reporter constructs under the control of the M2 and ML candidate CRMs revealed consistent patterns (Fig. 2*G–I* *;* Fig. 3; and not shown) and in both cases eGFP expression was restricted to nuclei, as expected by the presence of a nuclear localization signal in the vector.

**Figure 4:**
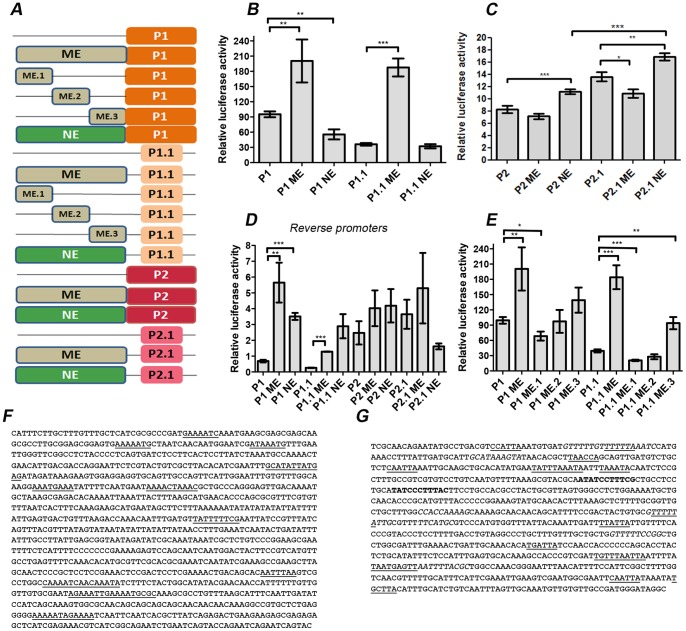
ME and NE boost P1 and P2 promoter activity, respectively. (A) Schematic representation of the Firefly luciferase reporter constructs used to transiently transfect *Drosophila* S2 cells. (B) ME, but not NE, potentiates P1 or P1.1 promoter activity. Basal promoter activity of P1 is significantly higher than P1.1 in these assays. Conversely, NE, but not ME, weakly enhanced P2 or P2.1 promoter function (C), being the relative luciferase activity measured approximately one tenth of that from P1 or P1.1. (D) Reversed P1 and P1.1 promoters still responded to ME and NE, although at much lower levels, whereas P2 and P2.1 did not significantly change reporter expression. (E) ME (GenBank accession number KJ201027) was subdivided into three smaller regions of approximately 1 kb (ME.1, ME.2 and ME.3). ME.3 retained most of ME ability to boost expression, although only for P1.1 it reached statistical significance. ME.3 and NE (GenBank accession number KJ201028) sequences are enriched in consensus binding sites for Mef2 (underlined) (*F*) and nervous system (*G*) transcription factors according to the following code: Hunchback sites, underlined; Ladybird early and Ladybird late, bold; Krüppel, italics respectively. Predictions used the Jaspar and rVista programs.

We have previously shown that Muscleblind is localized in the nuclei of embryonic pharyngeal, visceral and somatic muscles, in the larval photoreceptor system, and in repeated clusters of cells within the central nervous system [2], [3] (Fig. 2*B,C*). In combination with the *Hsp70* promoter, M2 drove robust eGFP expression in the somatic musculature of late embryos, approximately starting from stage 13. Notably, no eGFP expression was detected in other muscle derivatives or tissues where endogenous *muscleblind* is normally detected, particularly the CNS (Fig. 2*I*). As control, we generated transgenics carrying a promoter-less M2:eGFP construct, which revealed no eGFP expression (Fig. 2*D–F*), thus confirming that M2 had no promoter activity by itself but requires the presence of a promoter to exert its enhancer activity. Similarly, double eGFP and Muscleblind immunostaining of fly embryos carrying ML:eGFP constructs revealed that ML drove expression to clusters of cells in the ventral cord of late embryos that overlapped with those expressing endogenous Muscleblind (Fig. 3). First signal started at developmental stage 12 and no eGFP expression was detected in tissues other than the CNS. Consistently, ML included predicted binding sites for factors involved in nervous system development such as Ladybird early (Lbe), Ladybird late (Lbl), Krüppel (Kr) and Hunchback (Hb) [40], [41], [42], [43] (Fig. 4*G*). In summary, these results support that M2 and ML are somatic muscle and CNS-specific enhancers of *muscleblind,* respectively, at least during embryonic development. We therefore renamed these candidate CRMs as “ME” (from muscle enhancer) and “NE” (from nervous system enhancer). Importantly, both ME and NE were capable of activating a heterologous promoter, a typical ability of transcriptional enhancer elements.

### 4. Characterization of the Muscle and Nervous Enhancer Elements of *muscleblind*

To test the ability of ME and NE genomic regions to enhance transcription from the putative *muscleblind* promoters, we used luciferase reporter assays in *Drosophila* S2 cells. We used S2 cells because this is a well characterized cell line whereas muscle or neuron-specific cell lines were not immediately available. Both enhancer regions were cloned upstream to each of the putative promoters P1, P1.1, P2 and P2.1 in their forward (Fig. 4A) and reverse (not shown) orientations in the *pGL3* basic vector, which carries the Firefly luciferase reporter. As controls, ME and NE were tested in promoter-less constructs and no luciferase activity was detected (data not shown), thus confirming that the enhancer regions do not have any transcriptional activity by themselves. When promoters were in the forward orientation we observed that the transcription originated from P1 and P1.1 was strongly enhanced by ME, whereas NE had no effect on P1.1, or even decreased transcription when in combination with P1 (Fig. 4*B*). Similarly, P2 and P2.1 promoter activity was significantly enhanced by NE (around 30% and 20%, respectively), but remained unchanged when in combination with ME that even repressed transcription from P2.1 (Fig. 4*C*). As control of promoter directionality, luciferase levels of constructs carrying promoters in their reverse orientation were measured. Consistently, relative luciferase readings dropped to close to background levels in constructs containing promoters in their reverse orientation (Fig. 4 compare *B,C* with *D*), although both ME and NE still managed to significantly potentiate transcription from P1 and P1.1. Thus, promoter activity is orientation-dependent, as reversed promoters expressed significantly less luciferase reporter, and the activity of the ME and NE enhancers on P1 and P2 suggests enhancer-promoter communication specificity.

ME function was further analyzed to narrow down sequences necessary for enhancer activity. This involved testing three smaller regions, approximately 1 kb each, here referred to as ME.1, ME.2 and ME.3 according to their relative position in the original region (Fig. 4*A*). These sequences were cloned upstream to the P1 and P1.1 promoters and were used in luciferase reporter assays in S2 cells (Fig. 4*E*). Compared to the luciferase activity of the promoter alone, ME.3 was the only subregion able to significantly increase transcription from P1.1, also showing the same trend on the P1 promoter. Notably, all other subregions tested either did not boost expression from P1 or P1.1 or even inhibited it. Therefore, these data, and bioinformatics analyses support that ME.3 contains sequence motifs necessary for ME enhancing activity, including consensus Mef2 binding sites (Fig. 4*F*), but they also suggest that for maximum enhancing activity all three subregions are required. Bioinformatics analyses in NE found an enrichment of targets for nervous system transcription factors (Fig. 4*G*).

### 5. Functional Conservation of Human MBNL1 Promoter

**Figure 5:**
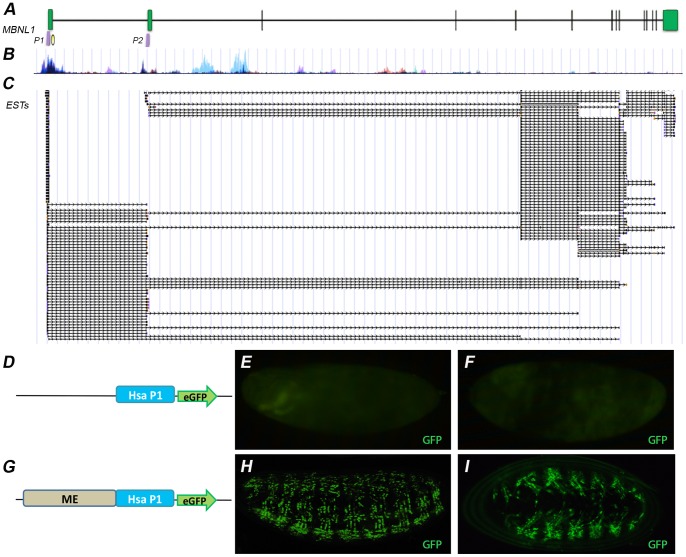
Genomic organization of the human MBNL1 gene. (A) Scale representation of 198 kb of the human MBNL1 locus. Green boxes correspond to exons and black lines to introns. Tested promoter regions are indicated as P1 and P2; a yellow circle denotes a predicted CpG island. (B) Schematic representation of H3K27Ac marks, typical of promoter regions, on seven human cell lines. (C) 5′-Ends of ESTs mapping to the MBNL1 locus support two potential transcription start sites for the gene. Data according to the UCSC Genome Browser [58]. (E,F,H,I) Direct visualization of the eGFP reporter under the control of the ME enhancer. (D–F) Enhancer-less Hsa-P1 construct is a negative control. (G–I) Flies carrying the ME enhancer upstream of the human MBNL1 promoter (Hsa-P1) reproduce Muscleblind expression in the somatic musculature. (E,H) Lateral and (F,I) ventral views of late embryos. All micrographs were taken at 200× magnification. Anterior is to the left and dorsal up, unless otherwise stated.

Sequence conservation between *Drosophila* and human MBNL1 promoter sequences was patently non-existent. However, analysis of available cDNA sequences and ESTs in the *MBNL1* locus suggested that human *MBNL1* might also use TSS located in exon 1 and in exon 2 (Fig. 5*A*). Consistently, putative TSS includes promoter marks such as CpG islands and histone modification tracks (Fig. 5*A,B*). We defined 500 bp around the predicted start region from both exons to test them as putative human MBNL1 promoters; Hsa-P1 in exon 1 (chr3: 151985544–151985045) and Hsa-P2 in exon 2 (chr3: 152016823–152017382). Synthetic Hsa-P1 and Hsa-P2 sequences were designed to replace the *Hsp70* promoter in the *Drosophila* transformation pH-Stinger vector. No eGFP expression was observed in transgenic fly embryos carrying any of the human promoters alone (data not shown and Fig. 5*D–F*). However, we observed a robust expression of eGFP in the somatic musculature of embryos when ME drove expression of the Hsa-P1 promoter (Fig. 5*G–I* compare to Fig. 2*B,C*). This expression was not observed in similar reporter constructs where Hsa-P2 replaced Hsa-P1 (ME-Hsa-P2; not shown). These data support that Hsa-P1 can initiate transcription, as we have also demonstrated for the *muscleblind* P1 promoter, and that ME is a muscle enhancer on a variety of promoters.

## Discussion

*Muscleblind* orthologues have attracted intense research interest due to their important role in vertebrate muscle development as well as involvement in several degenerative RNA-mediated diseases including DM1 and DM2, Huntingtońs disease, Huntington’s disease-like (HDL2) or spinocerebellar ataxia 8 (SCA8) [6], [44], [45], [46]. More recently, MBNL proteins were found to repress embryonic stem cell alternative splicing patterns, uncovering an additional role in the control of the cell pluripotency [22]. Despite this, little is known about the transcriptional regulation of *muscleblind* genes both in *Drosophila* and in vertebrates.

As a means of dissecting the *cis*-regulation of *muscleblind*, we analyzed a genomic DNA fragment harboring the *muscleblind* locus and its upstream region, looking for CRMs that regulate basal initiation of transcription (promoters) and tissue-specific expression (enhancers). *In silico* and *in vivo* studies identified two putative promoters, P1 in exon 1 and P2 in exon 2, and two intronic tissue-specific regulatory elements, a region of 3340 bp which drives specific expression in somatic muscle (ME) and a region of 830 bp which drives expression in central nervous system (NE). Both enhancers had been selected because of their enrichment in Mef2 binding sites according to ChIP-on-chip data [37], because Mef2 is a known positive regulator of *muscleblind* in the embryo [2]. Nevertheless, other enhancer elements must exist in order to explain the rich embryonic expression pattern of *muscleblind,* which also includes expression in visceral and pharyngeal musculature, the Bolwiǵs organ (the larval photoreceptor system) and the imaginal discs [2], [3]. Regarding this, putative CRMs that did not reproduce any embryonic pattern in our study can not be discarded as functional in other developmental stages.

Enhancer regions ME and NE were able to boost expression originating from the heterologous *Hsp70* promoter in transgenic embryos. However, in luciferase S2 assays, ME preferentially activated the P1 promoter while NE showed preference for P2, although the enhancer activity of NE was smaller than that of ME and transcription arising from P2 was on average one tenth of that from P1 (Fig. 4*B,C*). Our results in transgenic flies carrying the ME in combination with the human *MBNL1* P1 promoter also suggest enhancer promoter specificity as ME only induced reporter expression from Hsa-P1, but not from Hsa-P2. The use of alternative promoters is a known mechanism of transcriptional regulation, which has been reported to influence levels of transcription, turnover or translation efficiency of mRNA isoforms with different leader exons, tissue specificity [47] and to generate protein isoforms differing in the amino termini (reviewed in [48]). Furthermore, different core promoters have been found to possess distinct regulatory activities driven by the same enhancer in the *Drosophila* embryo ([49]). In *muscleblind*, the potential *in vivo* use of P1 and P2 as alternative promoters would have no consequences as for the encoded protein since the start codon is located in exon 2, which is downstream of both. However, the *in vivo* relevance of the internal promoter P2 remains to be specifically addressed. It is also worth mentioning that whereas the identified enhancers provided strong activation of the reporter *in vivo*, particularly ME, the measured activation in S2 cells was discrete, reaching some 6-fold increase over the promoter alone condition (Fig. *4B*). This may stem from the particular combination of transcription factors that S2 cells express, which may not be particularly favourable to activate myogenic enhancers. Mef2, for example, is weakly expressed in S2 cells according to modENCODE data [*50*], and, consistently, Muscleblind is only barely detectable in this cell line [51]. Nevertheless, the low expression of Muscleblind in S2 cells offers an opportunity to test the activating potential of candidate regulatory transcription factors.

Despite that sequences homologous to ME or NE in human *MBNL1* are not obvious, ENCODE Chip-seq data confirms that there is a high concentration of transcription factor binding sites in the first intron of *MBNL1*, including multiple MEF2A and MEF2C binding regions, thus suggesting that MEF2, a central regulator of diverse developmental programs [52], is also involved in the regulation of human *MBNL1* transcription. Indeed, detailed information on the multiple transcription factors converging on the *Drosophila* ME and NE enhancer elements would help in the identification of the functionally equivalent enhancer regions that integrate inputs from the same factors in humans. Although hypothetical, the functional conservation among fly and human *muscleblind* enhancers is conceivable. *Deformed* enhancers, for example, drive meaningful spatial expression patterns *in vivo* in a mouse context [53]. In any event, this initial characterization of *cis*-regulatory regions is the first step towards the understanding of the transcriptional regulation of *muscleblind*.

## Materials and Methods

### Constructs

P1 and P2, and their shorter versions P1.1 and P2.1, promoter regions were synthesized by GenScript with *Nhe*I/*Xho*I terminal adapters and were provided cloned into the *pUC57* vector. High fidelity PCR (KAPA HiFi DNA Polymerase, KAPA biosytems) was used to subclone into the *pGL3-Basic* vector (Promega) previously linearized with the same enzymes. ME, ME.1, ME.2, ME.3 (spanning from 13170622–13171543, ME.1; 13171544–13172759, ME.2; 13172760–13173908, ME.3) and NE were obtained from *Drosophila* genomic DNA by high fidelity PCR and were cloned into *Kpn*I/*Sac*I digested *pGL3-Basic* vectors already including different promoter regions. To generate transgenic flies, M1, M2, M3, H and ML fragments were PCR amplified from genomic DNA and were cloned into the *Bgl*II and *Xba*I sites of the *Drosophila* expression vector pH-Stinger [54]. Synthetic Hsa-P1 and Hsa-P2 promoter regions, including *Pst*I, *Bgl*II, *Xho*I (5′) and *Hind*III, *Pst*I (3′) adapter sites, were cloned into the *pUC57* vector and subsequently transferred to the *Pst*I site of the pH-Stinger vector. To generate ME-Hsa-P1 and ME-Hsa-P2 constructs, M2 was amplified with specific oligos containing *Bgl*II/*Xho*I adapters, digested, and cloned into the corresponding sites of the pH-Stinger vector already containing the candidate human promoters. All constructs were confirmed by sequencing. Description of used primers is in Table 2.

**Table 2:** Sequence and melting temperature (Tm) of PCR primers used for cloning genomic DNA.

Primer pair	Forward 5′→3′	Tm	Reverse 5′→3′	Tm
**M1 pH-Stinger**	GA**AGATCTC**GGTTCGACGTGACTTTTGC	70,1	GC**TCTAGA**TAATAATAGATAATAAATATGG	52.2
**M2 pH-Stinger**	GA**AGATCT**CTCGACAAACAATTGTCAG	66.8	GC**TCTAGA**TGCCGATGACGTTTCAAT	70.6
**M3 pH-Stinger**	GA**AGATCT**TGAGTTTCGGTCTGATGCC	66.9	GC**TCTAGA**ATGCATTTCAAATTTTGG	62.0
**H pH-Stinger**	GA**AGATCT**ACTCTTTGTCTATTTTCAC	52.3	GC**TCTAGA**CATGCCATTGTGGAAAAGC	67.5
**ML pH-Stinger**	GA**AGATCT**TAACCAGCAGTTGATGTCT	65.5	GC**TCTAGA**TTTGCCTATCCCATCGGCA	74.2
**P1 pGL3-Basic**	ATT**GCTAGC**TTGAAGTTACTAAA	55.9	ATT**CTCGAG**GAACGACTTTCGACA	68.9
**P1.1 pGL3-Basic**	ATT**GCTAGC**GCAGAGAGTGAAAGA	67.2	ATT**CTCGAG**GTGACT GAGCAGAA	66.3
**P2 pGL3-Basic**	ATT**GCTAGC**TTGCATTCTGCATAA	65.5	ATT**CTCGAG**GGTTTTGAGTGCTCC	69.3
**P2.1 pGL3-Basic**	ATT**GCTCGC**ATTTCTATTTATCTC	56.6	ATT**CTCGAG**CCTCTCGCGACAC	71.0
**ME pGL3-Basic**	TAT**GGTACC**CTCGACAAACAATTGTCAG	69.9	ATT**GAGCTC**TGCCGATGACGTTTCAAT	73.4
**NE pGL3-Basic**	TAT**GGTACC**TAACCAGCAGTTGATGTCT	67.2	ATT**GAGCTC**TTTGCCTATCCCATCGGCA	76.7
**ME.1 pGL3-Basic**	TAT**GGTACC**CTCGACAAACAATTGTCAG	69.9	ATT**GAGCTC**TAACGGTCGGCAAAGG	72.3
**ME.2 pGL3-Basic**	TAT**GGTACC**GTGTGCTGACGTCGTTAGG	74.1	ATT**GAGCTC**AAAGGCACAGGGTCC	72.0
**ME.3 pGL3-Basic**	TAT**GGTACC**GGTTGACAAACGATTCGG	75.7	ATT**GAGCTC**TGCCGATGACGTTTCAAT	73.4

Restriction sites are highlighted in bold.

### Cell Culture and Dual Luciferase Assays

*Drosophila melanogaster* Schneider 2 cells (S2) were cultured at 27°C in growing media containing 90% Schneideŕs insect media (Gibco), 10% heat inactivated fetal bovine serum (FBS), 100 units/ml of penicillin and 100 mg/ml of streptomycin. 48 h before transfection, 10^6 ^log-phase cells were transferred onto 24-well plates (300 μl per well). 4 μl of Cellfectin reagent (Invitrogen) in 200 μl of serum and antibiotic-free medium were used to co-transfect 450 ng of the pGL3 reporter plasmid of interest and 25 ng of *Renilla* luciferase. A GFP expressing vector served as transfection efficiency control. Cells were incubated 16 h with transfection mix, then media was replaced by Schneideŕs complete medium and the culture was additionally maintained 24 h at 27°C. Luciferase expression was monitorized using the Dual-Luciferase Reporter Assay System (Promega). Briefly, this involved adding 100 μl of lysis buffer per well, shaking for 15 min and transferring the lysate to a white 96-well plate with 40 μl of luciferase substrate. After 10 s of luminescence detection, Stop&Glo buffer was added and luminescence measurement was repeated. Luminescence readings used an EnVision plate reader (PerkinElmer). In cell culture luciferase reporter assays, all graphs show the average of three independent experiments with three technical replicates each. P-values were obtained using a two-tailed, non-paired t-test (α = 0.05). Welch’s correction was applied when variances were significantly different.

### Immunohistochemistry of *Drosophila* Embryos

Embryos were fixed for 20 min using 4% paraformaldehyde in PBS, devitelinized with a heptane:methanol 1∶1 mixture, and blocked with 0.1% Triton-X-100 in PBS (PBT) with 1% BSA for 15 min and later with 2% BSA in PBT for 30 min. Subsequently they were incubated with rabbit anti-GFP (1∶200 Torrey Pines Biolabs) antibody diluted in blocking solution containing 1% donkey serum for 2 h at room temperature. After washes embryos were incubated with an anti-rabbit-FITC (1∶200 Calbiochem) secondary antibody for 45 min. Muscleblind detection used sheep anti-Muscleblind (1∶500 [55]) for 2 h followed by washes and primary antibody recognition with sheep biotin-conjugated secondary antibody (1∶100, Sigma) for 2 h. Then, washed embryos were incubated with ABC solution (ABC kit, VECTASTAIN) for 30 min at room temperature, and were washed and incubated with streptavidin-Texas Red (1∶1000, Vector) for 45 min. In all cases embryos were washed 3× with 1% BSA in PBT and were mounted in Vectashield (Vector) with 2 μg/ml DAPI. Images were taken on an Olympus FluoView FV100 confocal microscope. At least 10–15 embryos of the desired stage, and showing the relevant expression patterns, were analyzed.

### *Drosophila* Strains and Transgenics

Genomic DNA was extracted from the *Drosophila* genome project strain *y^1^; Gr22b^1^ Gr22d^1^ cn^1^ CG33964^R4.2^ bw^1^ sp^1^; LysC^1^ MstProx^1^ GstD5^1^ Rh6^1^* (Bloomington *Drosophila* Stock Center, Bloomington IN). *y^1^w^1118^* flies were also from Bloomington IN. All constructs were injected into *w^1118^* embryos by BestGene, typically resulting in 2–6 independent transgenic lines.

### Web Resources

Predictions of transcription factor binding sites used JASPAR [56]. Phylogenetic conservation employed the Whole Genome rVISTA browser [57]. EST mapping was according to the UCSC Genome Browser database [58]. Predicted promoter consensus sequences used Suite for Computational Identification of Promoter Elements (SCOPE) [34]. Genomes of reference were *Drosophila* BDGP R5/dm3 and human GRCh37releases.

## References

[1] Kania A, Salzberg A, Bhat M, D’Evelyn D, He Y, et al (1995) P-element mutations affecting embryonic peripheral nervous system development in Drosophila melanogaster. Genetics 139: 1663–1678. 7789767 10.1093/genetics/139.4.1663 PMC1206492

[2] Artero R, Prokop A, Paricio N, Begemann G, Pueyo I, et al (1998) The muscleblind gene participates in the organization of Z-bands and epidermal attachments of Drosophila muscles and is regulated by Dmef2. Dev Biol 195: 131–143. 9520330 10.1006/dbio.1997.8833

[3] Begemann G, Paricio N, Artero R, Kiss I, Perez-Alonso M, et al (1997) muscleblind, a gene required for photoreceptor differentiation in Drosophila, encodes novel nuclear Cys3His-type zinc-finger-containing proteins. Development 124: 4321–4331. 9334280 10.1242/dev.124.21.4321

[4] Goers ES, Voelker RB, Gates DP, Berglund JA (2008) RNA binding specificity of Drosophila muscleblind. Biochemistry 47: 7284–7294. 18557632 10.1021/bi702252d PMC2706540

[5] Irion U (2012) Drosophila muscleblind codes for proteins with one and two tandem zinc finger motifs. PLoS One 7: e34248. 22479576 10.1371/journal.pone.0034248 PMC3315501

[6] Fernandez-Costa JM, Llamusi MB, Garcia-Lopez A, Artero R (2011) Alternative splicing regulation by Muscleblind proteins: from development to disease. Biol Rev Camb Philos Soc 86: 947–958. 21489124 10.1111/j.1469-185X.2011.00180.x

[7] Vicente-Crespo M, Pascual M, Fernandez-Costa JM, Garcia-Lopez A, Monferrer L, et al (2008) Drosophila muscleblind is involved in troponin T alternative splicing and apoptosis. PLoS ONE 3: e1613. 18286170 10.1371/journal.pone.0001613 PMC2238819

[8] Vicente M, Monferrer L, Poulos MG, Houseley JM, Monckton DG, et al (2007) Muscleblind isoforms are functionally distinct and regulate alpha-actinin splicing. Differentiation 75: 427–440. 17309604 10.1111/j.1432-0436.2006.00156.x

[9] Machuca-Tzili L, Thorpe H, Robinson TE, Sewry C, Brook JD (2006) Flies deficient in Muscleblind protein model features of myotonic dystrophy with altered splice forms of Z-band associated transcripts. Hum Genet 120: 487–499. 16927100 10.1007/s00439-006-0228-8

[10] Wang ET, Cody NA, Jog S, Biancolella M, Wang TT, et al (2012) Transcriptome-wide regulation of pre-mRNA splicing and mRNA localization by muscleblind proteins. Cell 150: 710–724. 22901804 10.1016/j.cell.2012.06.041 PMC3428802

[11] Picchio L, Plantie E, Renaud Y, Poovthumkadavil P, Jagla K (2013) Novel Drosophila model of myotonic dystrophy type 1: phenotypic characterization and genome-wide view of altered gene expression. Hum Mol Genet 22: 2795–2810. 23525904 10.1093/hmg/ddt127

[12] Salamov AA, Solovyev VV (2000) Ab initio gene finding in Drosophila genomic DNA. Genome Res 10: 516–522. 10779491 10.1101/gr.10.4.516 PMC310882

[13] Kanadia RN, Urbinati CR, Crusselle VJ, Luo D, Lee YJ, et al (2003) Developmental expression of mouse muscleblind genes Mbnl1, Mbnl2 and Mbnl3. Gene Expr Patterns 3: 459–462. 12915312 10.1016/s1567-133x(03)00064-4

[14] Miller JW, Urbinati CR, Teng-Umnuay P, Stenberg MG, Byrne BJ, et al (2000) Recruitment of human muscleblind proteins to (CUG)(n) expansions associated with myotonic dystrophy. Embo J 19: 4439–4448. 10970838 10.1093/emboj/19.17.4439 PMC302046

[15] Du H, Cline MS, Osborne RJ, Tuttle DL, Clark TA, et al (2010) Aberrant alternative splicing and extracellular matrix gene expression in mouse models of myotonic dystrophy. Nat Struct Mol Biol 17: 187–193. 20098426 10.1038/nsmb.1720 PMC2852634

[16] Osborne RJ, Lin X, Welle S, Sobczak K, O’Rourke JR, et al (2009) Transcriptional and post-transcriptional impact of toxic RNA in myotonic dystrophy. Hum Mol Genet 18: 1471–1481. 19223393 10.1093/hmg/ddp058 PMC2664149

[17] Masuda A, Andersen HS, Doktor TK, Okamoto T, Ito M, et al (2012) CUGBP1 and MBNL1 preferentially bind to 3′ UTRs and facilitate mRNA decay. Sci Rep 2: 209. 22355723 10.1038/srep00209 PMC3250574

[18] Adereth Y, Dammai V, Kose N, Li R, Hsu T (2005) RNA-dependent integrin a3 protein localisation regulated by the Muscleblind-like protein MLP1. Nature Cell Biology 7: 1240–1247. 16273094 10.1038/ncb1335 PMC2365307

[19] Rau F, Freyermuth F, Fugier C, Villemin JP, Fischer MC, et al (2011) Misregulation of miR-1 processing is associated with heart defects in myotonic dystrophy. Nat Struct Mol Biol 18: 840–845. 21685920 10.1038/nsmb.2067

[20] Charizanis K, Lee KY, Batra R, Goodwin M, Zhang C, et al (2012) Muscleblind-like 2-mediated alternative splicing in the developing brain and dysregulation in myotonic dystrophy. Neuron 75: 437–450. 22884328 10.1016/j.neuron.2012.05.029 PMC3418517

[21] Lin X, Miller JW, Mankodi A, Kanadia RN, Yuan Y, et al. (2006) Failure of MBNL1-dependent postnatal splicing transitions in myotonic dystrophy. Hum Mol Genet. 10.1093/hmg/ddl132 16717059

[22] Han H, Irimia M, Ross PJ, Sung HK, Alipanahi B, et al (2013) MBNL proteins repress ES-cell-specific alternative splicing and reprogramming. Nature 498: 241–245. 23739326 10.1038/nature12270 PMC3933998

[23] Lee KS, Cao Y, Witwicka HE, Tom S, Tapscott SJ, et al (2010) RNA-binding protein Muscleblind-like 3 (MBNL3) disrupts myocyte enhancer factor 2 (Mef2) {beta}-exon splicing. J Biol Chem 285: 33779–33787. 20709755 10.1074/jbc.M110.124255 PMC2962477

[24] Squillace RM, Chenault DM, Wang EH (2002) Inhibition of muscle differentiation by the novel muscleblind-related protein CHCR. Dev Biol 250: 218–230. 12297108 10.1006/dbio.2002.0798

[25] Lee KS, Squillace RM, Wang EH (2007) Expression pattern of muscleblind-like proteins differs in differentiating myoblasts. Biochem Biophys Res Commun 361: 151–155. 17644069 10.1016/j.bbrc.2007.06.165 PMC1994072

[26] Poulos MG, Batra R, Li M, Yuan Y, Zhang C, et al (2013) Progressive impairment of muscle regeneration in muscleblind-like 3 isoform knockout mice. Hum Mol Genet 22: 3547–3558. 23660517 10.1093/hmg/ddt209 PMC3736872

[27] Juni N, Yamamoto D (2009) Genetic analysis of chaste, a new mutation of Drosophila melanogaster characterized by extremely low female sexual receptivity. J Neurogenet 23: 329–340. 19169922 10.1080/01677060802471601

[28] Tabebordbar M, Wang ET, Wagers AJ (2013) Skeletal muscle degenerative diseases and strategies for therapeutic muscle repair. Annu Rev Pathol 8: 441–475. 23121053 10.1146/annurev-pathol-011811-132450

[29] Brook JD, McCurrach ME, Harley HG, Buckler AJ, Church D, et al (1992) Molecular basis of myotonic dystrophy: expansion of a trinucleotide (CTG) repeat at the 3′ end of a transcript encoding a protein kinase family member. Cell 69: 385. 10.1016/0092-8674(92)90418-c 1568252

[30] Liquori CL, Ricker K, Moseley ML, Jacobsen JF, Kress W, et al (2001) Myotonic dystrophy type 2 caused by a CCTG expansion in intron 1 of ZNF9. Science 293: 864–867. 11486088 10.1126/science.1062125

[31] Jiang H, Mankodi A, Swanson MS, Moxley RT, Thornton CA (2004) Myotonic dystrophy type 1 is associated with nuclear foci of mutant RNA, sequestration of muscleblind proteins and deregulated alternative splicing in neurons. Hum Mol Genet 13: 3079–3088. 15496431 10.1093/hmg/ddh327

[32] Fardaei M, Rogers MT, Thorpe HM, Larkin K, Hamshere MG, et al (2002) Three proteins, MBNL, MBLL and MBXL, co-localize in vivo with nuclear foci of expanded-repeat transcripts in DM1 and DM2 cells. Hum Mol Genet 11: 805–814. 11929853 10.1093/hmg/11.7.805

[33] Ohler U (2006) Identification of core promoter modules in Drosophila and their application in accurate transcription start site prediction. Nucleic Acids Res 34: 5943–5950. 17068082 10.1093/nar/gkl608 PMC1635271

[34] Carlson JM, Chakravarty A, DeZiel CE, Gross RH (2007) SCOPE: a web server for practical de novo motif discovery. Nucleic Acids Res 35: W259–264. 17485471 10.1093/nar/gkm310 PMC1933170

[35] de la Calle-Mustienes E, Feijoo CG, Manzanares M, Tena JJ, Rodriguez-Seguel E, et al (2005) A functional survey of the enhancer activity of conserved non-coding sequences from vertebrate Iroquois cluster gene deserts. Genome Res 15: 1061–1072. 16024824 10.1101/gr.4004805 PMC1182218

[36] Cunha PM, Sandmann T, Gustafson EH, Ciglar L, Eichenlaub MP, et al (2010) Combinatorial binding leads to diverse regulatory responses: Lmd is a tissue-specific modulator of Mef2 activity. PLoS Genet 6: e1001014. 20617173 10.1371/journal.pgen.1001014 PMC2895655

[37] Sandmann T, Jensen LJ, Jakobsen JS, Karzynski MM, Eichenlaub MP, et al (2006) A temporal map of transcription factor activity: mef2 directly regulates target genes at all stages of muscle development. Dev Cell 10: 797–807. 16740481 10.1016/j.devcel.2006.04.009

[38] Zinzen RP, Girardot C, Gagneur J, Braun M, Furlong EE (2009) Combinatorial binding predicts spatio-temporal cis-regulatory activity. Nature 462: 65–70. 19890324 10.1038/nature08531

[39] Barolo S, Castro B, Posakony JW (2004) New Drosophila transgenic reporters: insulated P-element vectors expressing fast-maturing RFP. Biotechniques 36: 436–440, 442. 10.2144/04363ST03 15038159

[40] De Graeve F, Jagla T, Daponte JP, Rickert C, Dastugue B, et al (2004) The ladybird homeobox genes are essential for the specification of a subpopulation of neural cells. Dev Biol 270: 122–134. 15136145 10.1016/j.ydbio.2004.02.014

[41] Jagla K, Georgel P, Bellard F, Dretzen G, Bellard M (1993) A novel homeobox nkch4 gene from the Drosophila 93E region. Gene 127: 165–171. 8099053 10.1016/0378-1119(93)90715-f

[42] Romani S, Jimenez F, Hoch M, Patel NH, Taubert H, et al (1996) Kruppel, a Drosophila segmentation gene, participates in the specification of neurons and glial cells. Mech Dev 60: 95–107. 9025064 10.1016/s0925-4773(96)00603-x

[43] Novotny T, Eiselt R, Urban J (2002) Hunchback is required for the specification of the early sublineage of neuroblast 7-3 in the Drosophila central nervous system. Development 129: 1027–1036. 11861485 10.1242/dev.129.4.1027

[44] Li LB, Bonini NM (2010) Roles of trinucleotide-repeat RNA in neurological disease and degeneration. Trends Neurosci 33: 292–298. 20398949 10.1016/j.tins.2010.03.004 PMC5720136

[45] Fiszer A, Krzyzosiak WJ (2013) RNA toxicity in polyglutamine disorders: concepts, models, and progress of research. J Mol Med (Berl) 91: 683–691. 23512265 10.1007/s00109-013-1016-2 PMC3659269

[46] Mykowska A, Sobczak K, Wojciechowska M, Kozlowski P, Krzyzosiak WJ (2011) CAG repeats mimic CUG repeats in the misregulation of alternative splicing. Nucleic Acids Res 39: 8938–8951. 21795378 10.1093/nar/gkr608 PMC3203611

[47] Bermingham JR Jr, Martinez-Arias A, Petitt MG, Scott MP (1990) Different patterns of transcription from the two Antennapedia promoters during Drosophila embryogenesis. Development 109: 553–566. 1976090 10.1242/dev.109.3.553

[48] Ayoubi TA, Van De Ven WJ (1996) Regulation of gene expression by alternative promoters. FASEB J 10: 453–460. 8647344

[49] Ohtsuki S, Levine M, Cai HN (1998) Different core promoters possess distinct regulatory activities in the Drosophila embryo. Genes Dev 12: 547–556. 9472023 10.1101/gad.12.4.547 PMC316525

[50] Roy S, Ernst J, Kharchenko PV, Kheradpour P, Negre N, et al (2010) Identification of functional elements and regulatory circuits by Drosophila modENCODE. Science 330: 1787–1797. 21177974 10.1126/science.1198374 PMC3192495

[51] Fernandez-Costa JM, Artero R (2010) A conserved motif controls nuclear localization of Drosophila Muscleblind. Mol Cells 30: 65–70. 20652497 10.1007/s10059-010-0089-9

[52] Landt SG, Marinov GK, Kundaje A, Kheradpour P, Pauli F, et al (2012) ChIP-seq guidelines and practices of the ENCODE and modENCODE consortia. Genome Res 22: 1813–1831. 22955991 10.1101/gr.136184.111 PMC3431496

[53] Awgulewitsch A, Jacobs D (1992) Deformed autoregulatory element from Drosophila functions in a conserved manner in transgenic mice. Nature 358: 341–344. 1353608 10.1038/358341a0

[54] Brand AH, Perrimon N (1993) Targeted gene expression as a means of altering cell fates and generating dominant phenotypes. Development 118: 401–415. 8223268 10.1242/dev.118.2.401

[55] Houseley JM, Wang Z, Brock GJ, Soloway J, Artero R, et al (2005) Myotonic dystrophy associated expanded CUG repeat muscleblind positive ribonuclear foci are not toxic to Drosophila. Hum Mol Genet 14: 873–883. 15703191 10.1093/hmg/ddi080

[56] Portales-Casamar E, Thongjuea S, Kwon AT, Arenillas D, Zhao X, et al (2010) JASPAR 2010: the greatly expanded open-access database of transcription factor binding profiles. Nucleic Acids Res 38: D105–110. 19906716 10.1093/nar/gkp950 PMC2808906

[57] Loots GG, Ovcharenko I, Pachter L, Dubchak I, Rubin EM (2002) rVista for comparative sequence-based discovery of functional transcription factor binding sites. Genome Res 12: 832–839. 11997350 10.1101/gr.225502 PMC186580

[58] Meyer LR, Zweig AS, Hinrichs AS, Karolchik D, Kuhn RM, et al (2013) The UCSC Genome Browser database: extensions and updates 2013. Nucleic Acids Res 41: D64–69. 23155063 10.1093/nar/gks1048 PMC3531082

